# Total Intracorporeal Robot Kidney Autotransplantation: Case Report and Description of Surgical Technique

**DOI:** 10.3389/fsurg.2020.00065

**Published:** 2020-12-11

**Authors:** Charles Van Praet, Edward Lambert, Liesbeth Desender, Benjamin Van Parys, Caroline Vanpeteghem, Karel Decaestecker

**Affiliations:** ^1^Department of Urology, Ghent University Hospital, Ghent, Belgium; ^2^Department of Thoracic and Vascular Surgery, Ghent University Hospital, Ghent, Belgium; ^3^Department of Anesthesiology, Ghent University Hospital, Ghent, Belgium

**Keywords:** kidney autotransplantation, kidney transplantation, robotics, robotic surgery, minimal-invasive surgery, case report

## Abstract

**Introduction and Objectives:** Kidney autotransplantation can be performed in patients with complex renal or ureteral pathology not suitable for *in situ* reconstruction, such as renal vasculature anomalies, patients with proximal or long complex ureteral strictures, or complex oncological cases. Robot-assisted surgery allows for a high-quality vascular and ureteral anastomosis and faster patient recovery. Robot-assisted kidney autotransplantation (RAKAT) is performed in two phases: nephrectomy and pelvic transplantation. In-between, extraction of the kidney allows for vascular reconstruction or kidney modification on the bench and safe cold ischemia can be established. If no bench reconstruction is needed, total intracorporeal RAKAT (tiRAKAT) is feasible. One case report in Europe has been described; however, to our knowledge no surgical video is available.

**Methods:** A 58 year-old woman suffered from right mid- and distal ureteral stenosis following pelvic radiotherapy 10 years prior for cervical cancer. A JJ stent was placed, but she suffered from recurrent urinary tract infections, and ultimately a nephrostomy was placed. Renogram demonstrated 43% relative right kidney function. As her bladder volume was low following radiotherapy, no Boari flap was possible and the patient refused life-long nephrostomy or nephrectomy. Therefore, tiRAKAT was performed using the DaVinci Xi system.

**Results:** We describe our surgical technique including a video. Surgical time (skin-to-skin) was 5 h and 45 min. Warm ischemia time was 4 min, cold ischemia 55 min, and rewarming ischemia 15 min. The abdominal catheter and bladder catheter were removed on the first and second postoperative day, respectively. The JJ stent was removed after 4 weeks. The patient suffered from pulmonary embolism on the second postoperative day, for which therapeutic low molecular weight heparin was started. No further complications occurred during the first 90 postoperative days. After 7 months, overall kidney function remained stable, right kidney function dropped non-significantly from 27 to 25.2 mL/min (−6.7%) on renal scintigraphy.

**Conclusion:** We demonstrated feasibility and, for the first time, a surgical video of tiRAKAT highlighting patient positioning, trocar placement, and intracorporeal cold ischemia technique.

## Background

Kidney autotransplantation (KAT) can be performed in patients with complex renal or ureteral pathology not suitable for *in situ* reconstruction, such as renal vasculature anomalies, patients with proximal or long complex ureteral strictures, or complex oncological cases. KAT is performed in two stages: a nephrectomy and reimplantation phase. In-between, the kidney is usually prepared on a bench to establish cold ischemia and to perform renal surgery, e.g., vascular reconstruction, lithiasis extraction, or tumor excision. The classic open approach requires either a midline puboxyphoid incision or both a flank and pelvic incision for nephrectomy and reimplantation, respectively. The associated morbidity and long convalescence limited widespread adoption of KAT (1). Adoption of minimal-invasive approaches in kidney transplantation have renewed interest in KAT (2). With laparoscopic nephrectomy before open reimplantation, the kidney can be removed and reimplanted through 1 pelvic incision, thus reducing morbidity (3). Furthermore, both nephrectomy and reimplantation can be performed robot-assisted, with only one peri-umbilical mini-laparotomy for a GelPOINT^®^ (AppliedMedical Resources Corp, Rancho Santa Margarita, CA, USA) trocar to remove and re-introduce the kidney (4). If no bench surgery of the kidney or renal vessels is needed and cold ischemia can be established intra-abdominally, total intracorporeal robot-assisted KAT (tiRAKAT) is feasible, as was demonstrated in 3 case reports so far (5–7). In this report we describe our first case of tiRAKAT. *To the best of our knowledge, this is the first reported operative video demonstrating surgical steps of the technique. It is also the first procedure performed with the Da Vinci Xi system*^®^ (Intuitive Surgical, Sunnyvale, CA, USA) *with demonstration of specific steps, e.g., simple redocking with boom rotation and integrated table motion during surgery. Furthermore, we highlight benefits and limitations of this technique and discuss the indications for either intracorporeal autotransplantation or autotransplantation with ex vivo bench reconstruction*.

## Case Presentation

**Figure 1:**
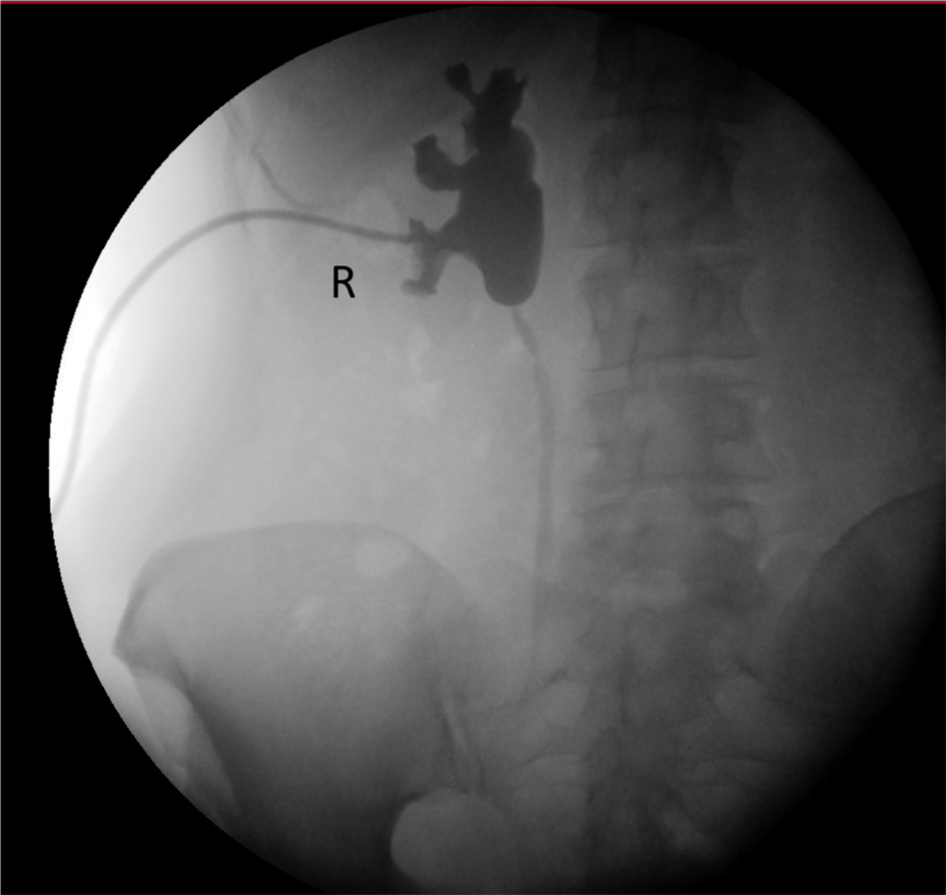
Pre-operative pyeloureterography demonstrating right-side mid-ureteral stricture at the level of the sacro-iliac joint.

Our patient is a 58 year-old woman who underwent curative pelvic radiotherapy and chemotherapy for stage T2bN1 cervical cancer in 2009. In March 2018, she presented in another hospital with macroscopic hematuria. Abdominal computed tomography showed hydronephrosis of the right kidney. Cystoscopy showed radiocystitis, ureteroscopy was not possible because the right ureter was too narrow, and a JJ stent was placed. Second-look flexible ureteroscopy showed a very narrow and stiff distal half of the ureter with no intraluminal lesions, and the stent was removed. She was then frequently monitored for 6 months. Right kidney hydronephrosis was progressive and kidney function deteriorated for which she was treated with a JJ stent. She consulted our hospital in January 2019 because of recurrent urinary tract infections. Ultrasonography showed persistent grade 2 hydronephrosis of the right kidney despite the JJ stent. Kidney function was normal with a right relative function of 43% on dimercaptosuccinic acid (DMSA) renal scintigraphy. The stent was removed, a nephrostomy was placed, and combined antegrade/retrograde ureterography demonstrated a ureteral stricture of about 50% of the right distal ureter (picture not available). Figure 1 presents a later antegrade ureterography showing a patent proximal half of the right ureter with no contrast in the distal half of the ureter, indicating stenosis. The following therapeutic options were considered:

1. Ureter reimplantation with Boari flap, however following pelvic radiotherapy she had a small bladder capacity. This could impair creation of a proper ureterovesical anastomosis without traction and could further decrease her functional bladder capacity.
2. Ileal bowel interposition, however this induces a risk of urinary obstruction by mucus, stone formation, metabolic abnormalities, and renal function deterioration (8).
3. Ureteroplasty with buccal mucosa was contra-indicated as this concerns too long a stricture in an irradiated graft bed (9).
4. Nephrostomy tube for life with 6-weekly changes in the outpatient clinic.
5. Right nephrectomy.
6. Right kidney autotransplantation.

The first two options were not advised by the treating urologist (KD) because this could deteriorate her quality of life. The patient did not want a nephrostomy tube any longer. She also wanted to retain both her kidneys if possible. Therefore, through shared-decision making, she opted for autotransplantation. Surgery was performed in September 2019 at Ghent University Hospital by KD, who is experienced in robotic uro-oncological surgery and kidney allo- and autotransplantation (4, 10).

### Surgery

#### Patient Preparation (Figure 2) and Trocar Positioning (Figure 3)

Surgery was performed with the DaVinci Xi^®^ (Intuitive Surgical, Sunnyvale, CA, USA) system. The patient was positioned on a PinkPad^®^ (Kebomed, Apeldoorn, The Netherlands) in dorsal decubitus on a Trumpf Medical^®^ operating table with integrated table motion. By inflating two pressure bags, positioned below the PinkPad^®^, one each under the ipsilateral pelvis and scapula, the patient was tilted into a 50° modified flank position. After strapping the patient to the table, the table was tilted 15° to obtain a 65° flank position for the nephrectomy phase. A small side support was put in front of the left anterior superior iliac spine to prevent the patient from slipping from the operating table.

**Figure 2:**
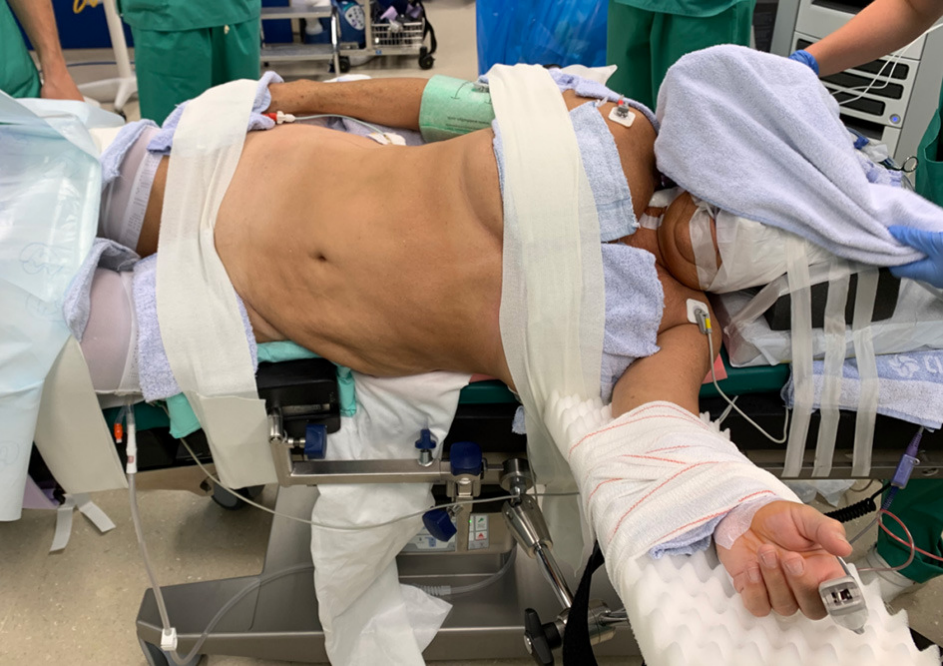
Patient positioning. The patient is positioned supine on a PinkPad^®^ (Kebomed, Apeldoorn, The Netherlands) with two pressure bags (one underneath the right pelvis and one underneath the right scapula) which are inflated. The patient is strapped to the table, which is tilted 15 degrees to obtain a lateral flank position for nephrectomy. A small side support is put in front of the left anterior superior iliac spine. For transplantation, the pressure bags are released, the table is untilted, and Trendelenburg is added. Care is taken for the position of the head and to avoid traction on the brachial plexus while changing positions.

**Figure 3:**
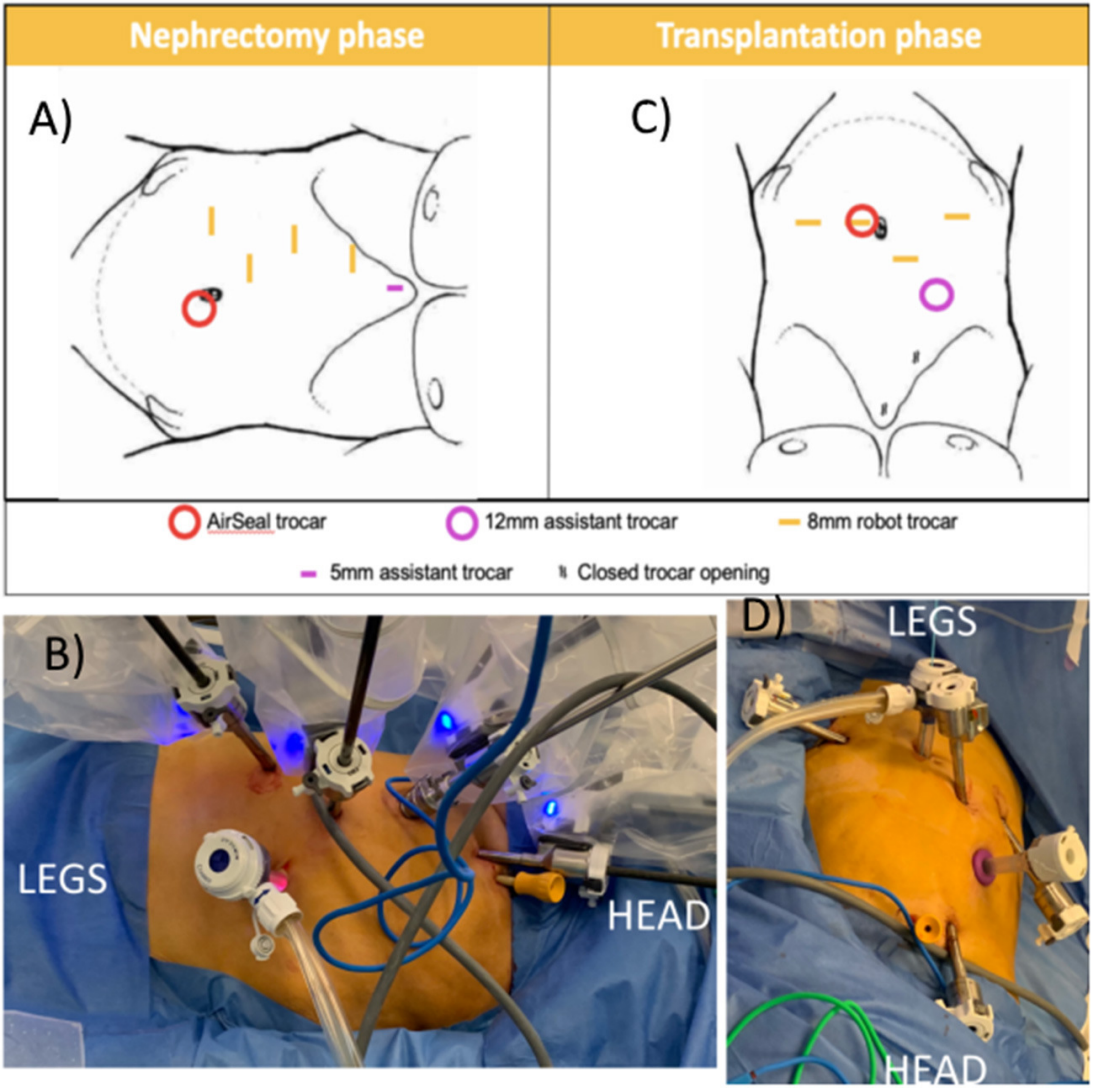
Trocar positioning using the Da Vinci Xi system. **(A,B)** Nephrectomy phase: patient positioned in modified flank position; four 8 mm robotic trocars in right hemi-abdomen, AirSeal^®^ (ConMed Corp, Utica, NY, USA) trocar as assistant trocar infra-umbilical and a 5 mm subxyphoid assistant port for liver retraction. **(C,D)** Transplantation phase: The two lowest 8 mm robotic trocars in the right hemi-abdomen are reused. A robotic trocar is placed inside the AirSeal^®^ trocar (port-in-port, not on photo) and an additional 8 mm robotic trocar is placed in the left lower abdomen. A 12 mm subcostal assistant trocar is placed, reusing a former robot trocar site. The most cranial robotic trocar and 5 mm port are no longer used.

The first 8 mm robotic port was inserted in the right hypochondrium using Hasson technique (11). Other trocars were inserted under direct vision as displayed in Figure 3: three additional 8 mm robotic ports, 1 assistant 12 mm AirSeal^®^ (ConMed Corp, Utica, NY, USA) port in the umbilicus, and one 5 mm assistant port subxyphoideal for liver retraction. Pneumoperitoneum was installed at 8 mm Hg pressure.

#### STEP 1 = Right Donor Nephrectomy (Nephrectomy Position)

During donor nephrectomy, the renal artery and vein were skeletonized to obtain maximal length. Fat was left on the kidney on several spots, including the lower pole, to allow intracorporeal manipulation. Before vessel clamping, the pelvis was inspected and severe radiofibrosis of the external iliac artery and vein was encountered. Therefore, we decided to first switch to the pelvic position to thoroughly prepare these vessels and minimize renal ischemia time.

#### STEP 2 = Right Iliac Vessel Preparation (Pelvic Position)

The robot was undocked, the table was tilted in the other direction, and the pressure bags were partially deflated until the patient reached dorsal decubitus. Twenty degrees of Trendelenburg was added. One additional robot port was placed in the left fossa, a robot port was put inside the 12 mm assistant port at the umbilicus (port-in-port), and the robot port (arm 3 in nephrectomy phase) was changed for a 12 mm assistant port (Figure 3). The boom of the robotic patient cart was rotated 90° and the robot was redocked. After preparation of the iliac vessels and creation of a retroperitoneal pocket, we again switched to nephrectomy position.

#### STEP 3 = Intravascular Cold Ischemia (Nephrectomy Position)

The ureter was clipped above the stenosis. After administering 2.500 units of heparin intraveneously, the renal artery and vein were clipped using 2 and 1 Click'aV^®^ (Grena, Nottingham, UK) clips consecutively. After securing the renal vessels, 2.500 units of protamine were administered intraveneously. Artery and vein were incised and a 5F feeding tube was inserted through the 12 mm assistant port into the renal artery. The artery was flushed with 4°C 0.9% sodium chloride solution. The kidney decolored fast and we saw clear effluent from the renal vein, indicating proper cold ischemia. The artery was completely transected and trimmed to prepare for reimplantation. The feeding tube was exchanged for an open-tip 5F Fogarty catheter with the balloon inflated with 0.5 mL water to block the catheter in the arterial lumen. Because of catheter luxation, the catheter was sutured to the artery and continuous irrigation with 4°C 0.9% sodium chloride solution was installed. A transfixing ligation of the clipped vascular stump was performed with Prolene 4.0 (Ethicon Inc., Johnson & Johnson Corp, Cincinnati, OH, USA) to prevent clip slipping. The kidney was placed in the pelvis and we converted back to pelvic position.

#### STEP 4 = Reimplantation (Pelvic Position)

**Figure 4:**
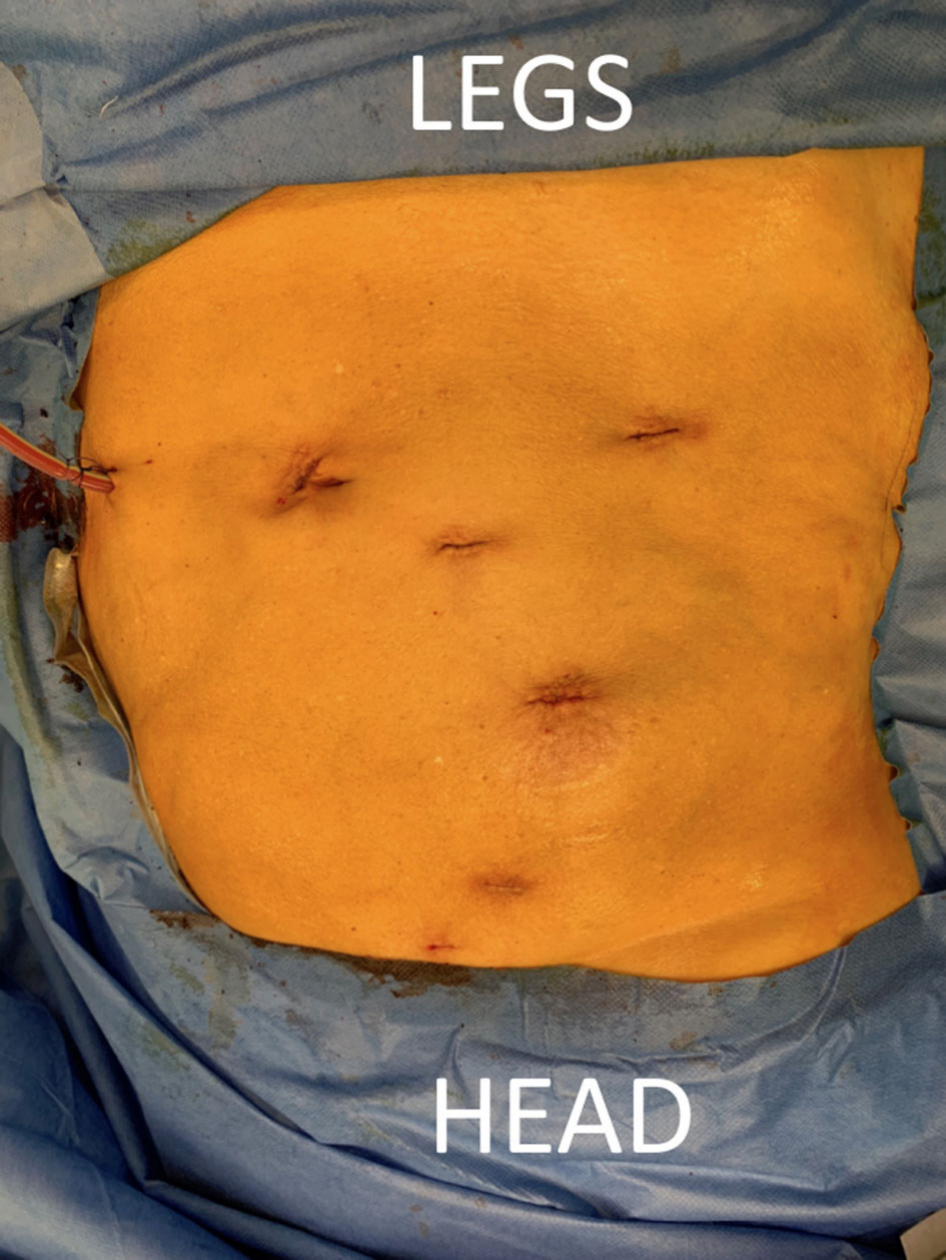
Final result.

Transplantation was performed according to the Vattikitu-Medanta technique as adopted by the European Association of Urology robot-assisted kidney transplantation group (2, 12, 13). Arterial flushing was continued until completion of the venous anastomosis and start of the arterial anastomosis. After completing the vascular anastomoses, the kidney was flipped in the retroperitoneal pocket, which was closed using clips. Diuresis from the transplant kidney was noted during surgery. The ureter was implanted posterolateral in the bladder following Lich-Gregoir technique over a JJ stent using a double armed PDS 5.0 (Ethicon Inc., Johnson & Johnson Corp, Cincinnati, OH, USA) continuous suture, without the need to drop the bladder and allowing easy cystoscopic ureter access in case of future interventions (14). Leakage test was negative. An abdominal drain was left in the pelvis (Figure 4).

Warm ischemia time (from renal artery clipping to intra-arterial cold perfusion) was 4 min. Cold ischemia time (from start cold perfusion to start arterial anastomosis) was 55 min. Rewarming ischemia time (from start arterial anastomosis to kidney reperfusion) was 15 min. Renal vein and artery anastomosis time was 5 and 13 min, respectively. Vesico-ureteral anastomosis time was 15 min. Total operation time (skin to skin) was 345 min. Robotic console time was 300 min. Estimated blood loss was 120 mL.

**Figure 5:**
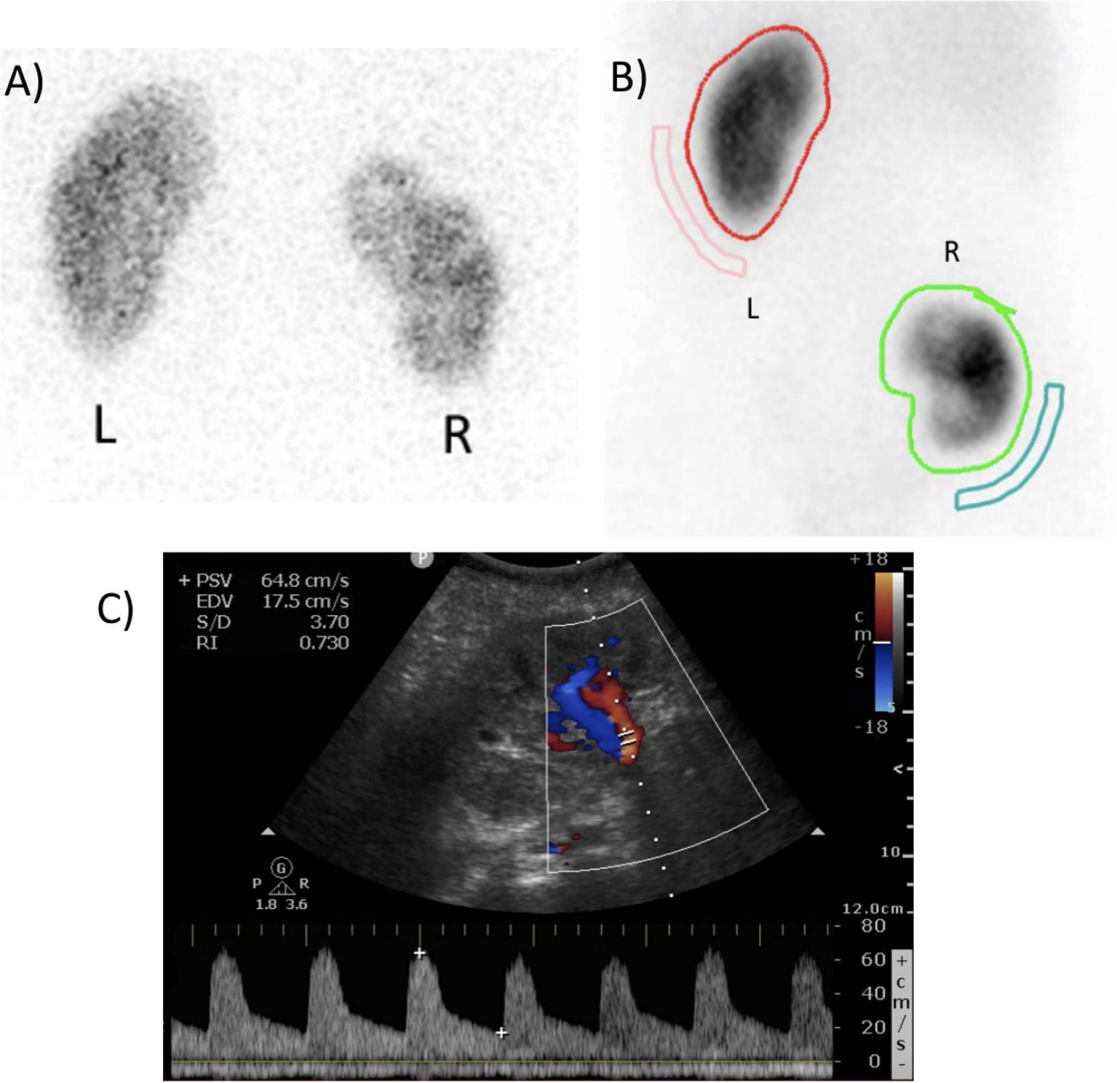
**(A)** Pre-operative dimercaptosuccinic acid (DMSA) renal scintigraphy showing a relative right kidney function of 43%. **(B)** Postoperative DMSA renal scintigraphy after 7 months, showing a relative autotransplant kidney function of 42%. **(C)** Postoperative duplex ultrasound of the autotransplant, demonstrating adequate perfusion with a resistance index of 0.730.

The evening before surgery, amoxicilline-clavulane acid was started based on pre-operative urine culture from the nephrostomy tube and continued for 5 days. Postoperatively, the nasogastric tube was removed immediately after extubation. The patient was monitored on intensive care until the next morning as is standard practice for kidney transplantation in our hospital. Doppler ultrasound of the transplant kidney immediately postoperative demonstrated a resistance index of 0.73, indicating adequate perfusion (Figure 5). Mean arterial blood pressure was maintained above 90 mmHg. The patient was at low risk for venous thromboembolism according to European Association of Urology (EAU) guidelines (15). Thrombo-embolic prophylactic stockings were applied peri-operatively and continued to be worn at least 2 weeks following surgery, low-molecular weight heparin in prophylactic dosage (enoxaparin sodium 40 mg) was started 8 h postoperatively and continued once daily. The patient was mobilized on the first postoperative day. The abdominal drain was removed on the first postoperative day. The bladder catheter was removed on the sixth postoperative day. The JJ stent was removed with flexible cystoscope under local analgesia 4 weeks postoperatively, and ultrasonography 1 week later showed no hydronephrosis.

Because of an unexplained drop in oxygen saturation on the second postoperative day, pulmonary arteriographic computed tomography was performed which demonstrated pulmonary embolism. Therapeutic anticoagulation was started and continued for 6 months (Clavien-Dindo grade 2) (16). Duplex ultrasonography revealed a deep venous thrombosis of the right popliteal vein. The patient already had chronic lymphedema of the right leg pre-operatively which remained stable postoperatively. No further complications were recorded during the first 3 months postoperatively.

Serum creatinine was 0.74 mg/dL pre-operatively, 0.97 and 0.83 mg/dL on the first and second postoperative day. Three and 6 months postoperatively, it was 0.80 and 0.72 mg/dL, respectively. Diethylenetriaminepentacetate (DTPA) and DMSA renal scintigraphy pre-operatively and 3 months postoperatively showed an absolute total kidney function of 63 and 59 mL/min and a relative right kidney function of 43 and 35%, respectively. This results in an absolute right kidney function dropping from 27 to 20.65 ml/min (−23.5%). However, at 7 months postoperatively, DTPA and DMSA renal scintigraphy demonstrated an absolute total kidney function of 60 mL/min and a relative autotransplant function of 42%, resulting in an absolute autotransplant function of 25.2 mL/min (non-significant drop of −6.7% compared to baseline, Figure 5).

After 7 months, the patient is doing well. She is happy to live without nephrostomy and she stopped anticoagulation 1 month ago. She has no residual symptoms from her pulmonary embolism and cardiac ultrasonography demonstrated normal right ventricular function.

## Discussion

Robotic surgery has renewed interest in KAT, with RAKAT emerging as an alternative treatment option to salvage the kidney in patients with complex renovascular, renal, or ureteral pathology [(10), Van Parys et al., in review]. Often, kidney extraction and bench surgery are necessary, e.g., for vascular reconstruction, lithiasis extraction, or tumor excision. However, with intra-arterial cooled perfusion, the kidney can remain intraperitoneally during the whole procedure if no bench surgery is necessary. For these selected patients, tiRAKAT is feasible to minimize postoperative morbidity. We described in detail the first case we performed. There are some specifics and future changes to our technique that we would like to stress below.

Limiting ischemia time is crucial in transplant surgery. All case reports of tiRAKAT show short warm, cold, and rewarming ischemia times (5–7). Especially our rewarming ischemia time of 15 min is shorter compared to robotic kidney allotransplantation, where median time is 50 min (interquartile range 38.5–61.5) (2). While rewarming ischemia time is only determined by the time to construct the arterial anastomosis, cold ischemia time depends on multiple factors: time to convert from flank to pelvic position and redock the robot, time to prepare the iliac vessels for reimplantation, and venous anastomosis time. The DaVinci Xi^®^ system with boom rotation allows for quick rotation of the robot arms while the patient cart remains stationary, which cannot be attained with the Si or X robotic systems. Also, integrated table motion allows for quick table movement without the need to undock the robot in case, e.g., more Trendelenburg is needed. Also, the Xi system has no dedicated camera port, so “port hopping” of the camera also reduces operative time. Gas insufflation using the AirSeal^®^ technique allows us to work under low intraperitoneal pressure, which is associated with less postoperative pain and better kidney perfusion (17, 18). With the described port placement, only 1 additional robot port in the contralateral fossa is needed for the transplantation phase. To reduce cold ischemia time, this port might already be placed during initial trocar positioning. Similarly, the second robotic trocar from the top can already be put through a 12 mm assistant trocar from the start of the surgery, as this becomes an assistant trocar during transplantation phase. With more cases performed, our technique is likely to evolve and become more time-efficient.

In future cases we will start intra-arterial cold ischemia with a Fogarty catheter instead of using a feeding tube first. We will also fix the catheter with a stitch to the renal artery instead of blocking it using balloon insufflation. First, the catheter with inflated balloon might slip during kidney manipulation, as was the case in our patient. Second, for balloon insufflation the catheter has to be inserted for a couple of centimeters into the renal artery to prevent luxation. A short renal artery or an early bifurcation may thus impede proper irrigation. The technique of intra-arterial cold ischemia was previously described in case reports of tiRAKAT (5–7), a series of KAT with laparoscopic nephrectomy and open reimplantation (19), a series of laparoscopic partial nephrectomy (20), and a recent series of robotic partial nephrectomy where a renal artery catheter is placed percutaneously by the interventional radiologist just before surgery (21).

TiRAKAT is only possible in cases where *ex vivo* bench surgery is not required. Ideally, the patient has a solitary renal artery and vein, although robotic allotransplantation with multiple anastomoses for multiple vessels has been described (22). An early branch of the renal artery is a contra-indication because proper intra-renal cooled perfusion of this branch is impeded. Although not yet performed, we believe that transplantation in the contralateral iliac fossa is feasible using our described technique.

We would like to stress that tiRAKAT should only be performed by surgeons with experience in robotic renal, vascular, and transplant surgery. Also, because of the specifics of KAT, we would suggest surgeons starting with robotic KAT to perform the first cases with a mini-laparotomy and GelPOINT^®^ for specimen extraction as described previously (4). This will allow proper cold ischemia on the bench. Once this procedure is mastered, the surgeon can proceed with a total intracorporeal approach. Also, during tiRAKAT, we prepare an “exit strategy”: in case of inadequate cold ischemia (e.g., no full discoloration of the kidney), an emergency mini-laparotomy should be performed to extract the kidney and establish proper cold ischemia on the bench.

Although we presume that morbidity is minimized using tiRAKAT, complications not associated with the mini-laparotomy remain unchanged. This was demonstrated in our patient who developed a deep venous thrombosis and pulmonary embolism, despite her being at low risk for venous thromboembolism according to EAU guidelines (15). We presume the thrombosis was provoked by manipulation and clamping of the external iliac vein and was not related to the total intracorporeal approach. Other factors that could have contributed were previous radiotherapy with radiofibrosis of the pelvic vessels and total operation time of almost 6 h. We believe that our operation time will go down with more experience.

Our patient had a drop in absolute autograft function (27 to 20.65 mL/min, −23.5%) after 3 months, however this recuperated to 25.2 mL/min 7 months postoperatively (−6.7% compared to baseline). As this difference is <20%, it is not considered significant (23). As we would not expect improvements in graft function over time, we hypothesize that this temporary drop is either patient-related (e.g., reduced fluid intake or nephrotoxic medication use) or related to the scintigraphy or its interpretation.

As this is only a single case, we can only state that this surgery is feasible and share our experience. Case series are necessary to demonstrate overall safety of the procedure, determine whether cold ischemia remains adequate throughout the whole procedure (e.g., by measuring temperature of the effluent of the renal vein), and assess long-term complications and kidney function as compared to RAKAT with mini-laparotomy.

## Conclusion

We demonstrate the feasibility and the first surgical video of a tiRAKAT. In patients without need for kidney, vascular, or ureteral reconstruction *ex vivo*, this approach may further limit operation time and minimize morbidity.

## Data Availability Statement

All datasets presented in this study are included in the article/Supplementary Material.

## Ethics Statement

The patient provided written consent for anonymous publication of this case report and accompanying images and video. The manuscript was constructed adhering to SCARE criteria for case reports (24).

## Author Contributions

CVP was the main author and video editor. KD performed surgery and carried out literature search. BV carried out literature search. All authors were part of the surgical team and critically revised the manuscript.

## Conflict of Interest

KD is a consultant for Intuitive Surgical, Sunnyvale, CA, United States of America. The remaining authors declare that the research was conducted in the absence of any commercial or financial relationships that could be construed as a potential conflict of interest.

## Supplementary Material

Supplementary Video 1 Totally intracorporeal robot-assisted kidney auto-transplantation. The video accompanying this article is protected by copyright held by KD.

## References

[1] Hardy JD Eraslan S. Autotransplantation of the kidney for high ureteral injury. J Urol. (1963) 90:563–74. 10.1016/S0022-5347(17)64454-9 14079696

[2] Breda A Territo A Gausa L Tugcu V Alcaraz A Musquera M. Robot-assisted kidney transplantation: the European experience. Eur Urol. (2018) 73:273–81. 10.1016/j.eururo.2017.08.028 28916408

[3] Fabrizio MD Kavoussi LR Jackman S Chan DY Tseng E Ratner LE. Laparoscopic nephrectomy for autotransplantation. Urology. (2000) 55:145. 10.1016/S0090-4295(99)00367-2 10754163

[4] Decaestecker K Van Parys B Van Besien J Doumerc N Desender L Randon C. Robot-assisted kidney autotransplantation: a minimally invasive way to salvage kidneys. Eur Urol Focus. (2018) 4:198–205. 10.1016/j.euf.2018.07.019 30093358

[5] Gordon ZN Angell J Abaza R. Completely intracorporeal robotic renal autotransplantation. J Urol. (2014) 192:1516–22. 10.1016/j.juro.2014.02.2589 24960467

[6] Lee JY Alzahrani T Ordon M. Intra-corporeal robotic renal auto-transplantation. Can Urol Assoc J. (2015) 9:E748–9. 10.5489/cuaj.3015 26664514 PMC4662430

[7] Doumerc N Beauval JB Roumiguie M Roulette P Laclergerie F Sallusto F. Total intracorporeal robotic renal auto-transplantation: a new minimally invasive approach to preserve the kidney after major ureteral injuries. Int J Surg Case Rep. (2018) 49:176–9. 10.1016/j.ijscr.2018.06.017 30015216 PMC6070672

[8] Tanagho EA. A case against incorporation of bowel segments into the closed urinary system. J Urol. (1975) 113:796–802. 10.1016/S0022-5347(17)59582-8 1152153

[9] Beysens M De Groote R Van Haute C Tailly T Lumen N Decaestecker K. Robotic lingual mucosal onlay graft ureteroplasty for proximal ureteral stricture. Eur Urol Suppl. (2018) 17:e1935. 10.1016/S1569-9056(18)32340-627115158

[10] Decaestecker K Territo A Campi R Van Parys B Bevilacqua G Desender L Robot-assisted kidney transplantation. In: Küçük S Canda AE editors. Medical Robots - New Achievements. London, UK: IntechOpen (2020).

[11] Hasson HM. Open laparoscopy: a report of 150 cases. J Reprod Med. (1974) 12:234–8. 4275895

[12] Menon M Sood A Bhandari M Kher V Ghosh P Abaza R Robotic kidney transplantation with regional hypothermia: a step-by-step description of the Vattikuti Urology Institute-Medanta technique (IDEAL phase 2a). Eur Urol. (2014) 65:991–1000. 10.1016/j.eururo.2013.12.006 24388099

[13] Gallioli A Territo A Boissier R Campi R Vignolini G Musquera M. Learning curve in robot-assisted kidney transplantation: results from the European Robotic Urological Society Working Group. Eur Urol. (2020) 78:239–47. 10.1016/S2666-1683(20)34238-5 31928760

[14] Gregoir W. Congenital vesico-ureteral reflux. Acta Urol Belg. (1962) 30:286–300. 13901619

[15] EAU Guidelines Edn. Presented at the EAU Annual Congress. Amsterdam (2020). ISBN 978-94-92671-07-3.

[16] Dindo D Demartines N Clavien PA. Classification of surgical complications: a new proposal with evaluation in a cohort of 6336 patients and results of a survey. Ann Surg. (2004) 240:205–13. 10.1097/01.sla.0000133083.54934.ae 15273542 PMC1360123

[17] Hua J Gong J Yao L Zhou B Song Z. Low-pressure versus standard-pressure pneumoperitoneum for laparoscopic cholecystectomy: a systematic review and meta-analysis. Am J Surg. (2014) 208:143–50. 10.1016/j.amjsurg.2013.09.027 24503370

[18] Sassa N Hattori R Yamamoto T Kato M Komatsu T Matsukawa Y. Direct visualization of renal hemodynamics affected by carbon dioxide-induced pneumoperitoneum. Urology. (2009) 73:311–5. 10.1016/j.urology.2008.09.047 19038429

[19] Ciancio G Gaynor JJ Sageshima J Chen L Roth D Kupin W. Favorable outcomes with machine perfusion and longer pump times in kidney transplantation: a single-center, observational study. Transplantation. (2010) 90:882–90. 10.1097/TP.0b013e3181f2c962 20703178

[20] Beri A Lattouf JB Deambros O Grull M Gschwendtner M Ziegerhofer J. Partial nephrectomy using renal artery perfusion for cold ischemia: functional and oncologic outcomes. J Endourol. (2008) 22:1285–90. 10.1089/end.2008.0152 18484894

[21] Liu F Yuan H Li X Ma X Wang M. Application of hypothermic perfusion via a renal artery balloon catheter during robot-assisted partial nephrectomy and effect on renal function. Acad Radiol. (2019) 26:e196–201. 10.1016/j.acra.2018.09.024 31284936

[22] Himanshu S Kishore T Vishnu R Challenges in robot-assisted kidney transplant: a case series of 22 patients. Videourology. (2020) 34 10.1089/vid.2019.0065

[23] Fleming JS Zivanovic MA Blake GM Burniston M Cosgriff PS British Nuclear Medicine S. Guidelines for the measurement of glomerular filtration rate using plasma sampling. Nucl Med Commun. (2004) 25:759–69. 10.1097/01.mnm.0000136715.71820.4a 15266169

[24] Agha RA Borrelli MR Farwana R Koshy K Fowler AJ Orgill DP. The SCARE 2018 statement: updating consensus Surgical CAse REport (SCARE) guidelines. Int J Surg. (2018) 60:132–6. 10.1016/j.ijsu.2018.10.028 30342279

